# Liver cancer in China: the analysis of mortality and burden of disease trends from 2008 to 2021

**DOI:** 10.1186/s12885-024-12334-2

**Published:** 2024-05-16

**Authors:** Yajun Qin, Changlin Tang, Jinhao Li, Jianping Gong

**Affiliations:** Department of Hepatobiliary Surgery, The Second Affiliated Hospital of Chongqing Medical University, Chongqing, 400010 People’s Republic of China

**Keywords:** Liver Neoplasms, Mortality, Burden of disease, PYLL

## Abstract

**Background:**

Liver cancer is one of the most common cancers in China. To understand the basic death situation and disease burden change trend, we analyze the death information of liver cancer among Chinese residents from 2008 to 2021.

**Methods:**

Data was collected from the Cause-of-Death Surveillance dataset of the National Cause-of-Death Surveillance System from 2008 to 2021. Excel 2016 was used for data entry and to calculate the Crude Mortality Rate (CMR), Age-Standardized Mortality Rate (ASMR), Potential Years of Life Lost (PYLL), and Potential Years of Life Lost Rate (PYLLR). SPSS 25.0 was used to statistically analyze CMR, ASMR, PYLL, and other indicators. Annual percent change (APC) and average APC(AAPC) was used for trend analysis and tested by t tests. Joinpoint 4.9.1.0 was used to calculate APC and AAPC. Age-Period-Cohort model was used to assess the effects of age, period, and birth cohort on liver cancer mortality.

**Results:**

From 2008 to 2021, 491,701 liver cancer deaths were reported in the National Disease Surveillance Points System. The ASMR of liver cancer in Chinese residents decreased from 27.58/100,000 in 2008 to 17.95/100,000 in 2021 at an average annual rate of 3.40% (t = -5.10, *P* < 0.001). The mortality rate was higher in males than in females (all *P* < 0.001) and higher in rural areas than in urban areas (all *P* < 0.001). The mortality rate of liver cancer varied significantly among eastern, central, and western China (all *P* < 0.001). The PYLLR of liver cancer in Chinese residents decreased from 2.89‰ in 2008 to 2.06‰ in 2021 at an average annual rate of 2.40% (t = -5.10, *P* < 0.001). Males had a lower PYLLR than females, decreasing at average annual rates of 2.20% (t = -5.40, *P* < 0.001) and 2.90% (t = -8.40, *P* < 0.001), respectively. Urban areas had a lower PYLLR than rural areas, decreasing at average annual rate of 3.30% (t = -4.00, *P* < 0.001) and 2.50% (t = -11.60, *P* < 0.001), respectively. Eastern, central, and western China decreased at average annual rates of 3.40%, 2.30%, and 2.10%, respectively (t = -7.80, -3.60, -7.10, *P* < 0.001 for all). The risk of China liver cancer mortality increased with age, decreased with birth cohort.

**Conclusions:**

The mortality and disease burdens of liver cancer in China decreased yearly and were higher in males and in people living in rural areas, with significant differences among those living in eastern, central, and western China.

**Supplementary Information:**

The online version contains supplementary material available at 10.1186/s12885-024-12334-2.

## Background

Liver cancer is one of the most common cancers in China. It is estimated that there were 905,700 new liver cancer cases and 830,200 new liver cancer deaths worldwide in 2020 [1]. There were 410,038 new liver cancer cases in China, accounting for 45.27% of the global total [2]. Liver cancer is the sixth most common cancer and the second leading cause of cancer-related death worldwide [3]. Meanwhile, China has a heavier disease burden of liver cancer. The GBD 2017 report shows that the DALYs caused by liver cancer in China reached 11,153.0 thousand in 2017, accounting for 53.7% of the global DALYs [4]. The acronym “DALY” stands for the disability-adjusted life year and is usually used to calculate the loss of healthy life years due to premature death and disability caused by diseases. DALYs can reflect the burden of diseases. The prognosis of liver cancer is also poor, and studies show that the 5-year survival rate in China is only 12.1% [5]. Liver cancer is characterized by a high incidence, high mortality, and heavy disease burden. This study was based on liver cancer mortality data from the Cause-of-death Surveillance dataset of the National Disease Surveillance System from 2008 to 2021. The aim was to analyze the trend of liver cancer mortality and disease burden by sex, urban‒rural areas, and regions and to provide references for liver cancer prevention and control.

## Materials and methods

### Material sources

The data on deaths caused by liver cancer were collected from the Cause-of-Death Surveillance dataset of the National Cause-of-Death Surveillance System from 2008 to 2021 [6]. The National Cause-of-Death Surveillance System is operated by the Chinese Center for Disease Control and Prevention. The International Classification of Diseases (ICD) code for liver cancer was ICD-10: C22. According to the regional division methods of the National Bureau of Statistics, China is divided into the eastern, central, and western regions. The eastern region includes Beijing, Tianjin, Hebei, Liaoning, Shanghai, Jiangsu, Zhejiang, Fujian, Shandong, Guangdong, and Hainan. The central region includes Shanxi, Jilin, Heilongjiang, Anhui, Jiangxi, Henan, Hubei, and Hunan. The western region includes Inner Mongolia, Guangxi, Chongqing, Sichuan, Guizhou, Yunnan, Tibet, Shaanxi, Gansu, Qinghai, Ningxia, and Xinjiang. According to the "Rules for the Compilation of Statistical Zoning Codes and Urban‒Rural Divisions," China is divided into urban and rural areas. The standard population comes from the Sixth National Census in 2010.

### Quality control

The National Cause-of-Death Surveillance System registers all deaths that occur in all jurisdictions. Quality control indicators: sex-ratio, the highest diagnostic unit, the highest diagnosis. Data quality evaluation: Example as 2021 cause-of-death detection dataset, all 605 surveillance sites reported data, involving 31 provinces, with a total population of 300 million, accounting for about 24% of the national population. Considering the accuracy and validity of the dataset, a total of 74 surveillance sites were excluded with a mortality rate of less than 4.5‰, and the data of 531 monitoring points were finally included. For the data quality control yearly, another dataset (Chinese Cancer Registry Annual Report, CCRAR) was introduced to evaluate the liver cancer mortality (The Result was in Table S1). According to Chinese guideline cancer registration (2016) and the standards of International Agency for Research on Cancer/International Association of Cancer Registries (IARC/IACR) on Cancer Incidence in Five Continents, Vol. XI, National Cancer Center have published a national criterion on data quality for Chinese cancer registration data, and valid data were included from 947 registries (covering 634,376,540 population) to form the CCRAR [7].

### Statistical analysis

Excel 2016 was used to record the different ages, sexes, urban‒rural areas, regional mortality rates (CMR and ASMR) and deaths of liver cancer in Chinese residents from 2008 to 2021, and to calculate PYLL and PYLLR. Joinpoint 4.9.1.0 was used to Calculate APC and AAPC. The joinpoint regression model uses the grid search method (GSM) to model and uses the Monte Carlo displacement test to select the model [8]. SPSS 25.0 was used for statistical analysis. The difference in mortality between different sexes, urban‒rural areas, and regions was compared by the χ2 test. The trend of mortality and PYLLR was described by APC and AAPC [9]. APC = 100% · (e.^β^-1) [10]. APC was tested by t test. ASMR is standardized based on data from the Sixth National Census in 2010. PYLL means the total years of life lost due to a certain cause of death in different age groups. PYLLR means the average loss of life per person in a certain area within a year. PYLL is calculated using the formula PYLL = Σa_i_·d_i_, where d_i_ is the deaths of age Group i, a_i_ is the remaining age, and a_i_ = 70-x_i_ + 0.5. X_i_ is the average age of the age Group i, and 0.5 is added when calculating a_i_ to eliminate the effect of imaginary age. PYLLR (‰) is calculated using the formula PYLL·1000/N, where N is the total number of people aged 1 ~ 70 in the survey population [11]. Compared with traditional mortality, PYLLR emphasizes the harmfulness of death in the young population, which can better reflect the overall picture of population death and disease burden. The higher the PYLLR, the greater the impact and burden of disease on human health. Based on the Poisson distribution, the Age-Period-Cohort model estimates the risk of disease in a certain population under the condition of adjusting for age, period and cohort factors. In this study, the Age-Period-Cohort web analysis tool (https://analysistools.cancer.gov/apc/) was used to analyze the effects of age, period, and cohort on liver cancer mortality, and tested by Waldtests [12].

## Results

### Mortality and trends of liver cancer

In 2008, the liver cancer CMR and ASMR in China were 25.52/100,000 and 27.58/100,000, respectively. In 2021, the CMR and ASMR were 24.92/100,000 and 17.95/100,000, respectively. The AAPCs of CMR and ASMR were -0.10% (t = -0.70, *P* = 0.47) and-3.40% (t = -5.10, *P* < 0.001), respectively. There was no significant difference in CMR trends, but there was a significant difference in ASMR trends.

In 2008, the liver cancer CMR and ASMR for males and females were 36.95/100,000, 41.16/100,000, 13.59/100,000, and 14.06/100,000, respectively. In2021, the liver cancer CMR and ASMR for males and females were 35.65/100,000, 27.37/100,000, 13.84/100,000, and 8.85/100,000, respectively. The AAPCs of liver cancer's CMR and ASMR in males and females were -0.50% (t = -0.60, *P* = 0.55), -3.30% (t = -7.2, *p* < 0.001), 0.30% (t = 1.60, *P* = 0.13), and -3.50% (t = -4.90, *P* < 0.001), respectively. There was no significant difference in the AAPC of liver cancer CMR and AMSR between males and females. From 2008 to 2021, the mortality rate of liver cancer in males was approximately 2–3 times higher than that in females (*P* < 0.001).

In 2008, the liver cancer CMR and ASMR in urban and rural areas were 22.98/100,000, 21.83/100,000, 26.89/100,000, and 31.32/100,000, respectively. In 2021, the liver cancer CMR and ASMR in urban and rural areas were 20.79/100,000, 14.57/100,000, 27.07/100,000, and 19.78/100,000, respectively. The AAPCs of liver cancer CMR and ASMR in urban and rural areas were -0.90% (t = -0.90, *P* = 0.35), -3.00% (t = -3.50, *p* < 0.001), -0.10% (t = -0.40, *P* = 0.72), and -3.20% (t = -12.5, *P* < 0.001), respectively. There was no significant difference in the AAPC of liver cancer CMR and AMSR between urban and rural areas. From 2008 to 2021, the mortality rate of liver cancer in rural areas was higher than that in urban areas (*P* < 0.001).

**Fig. 1:**
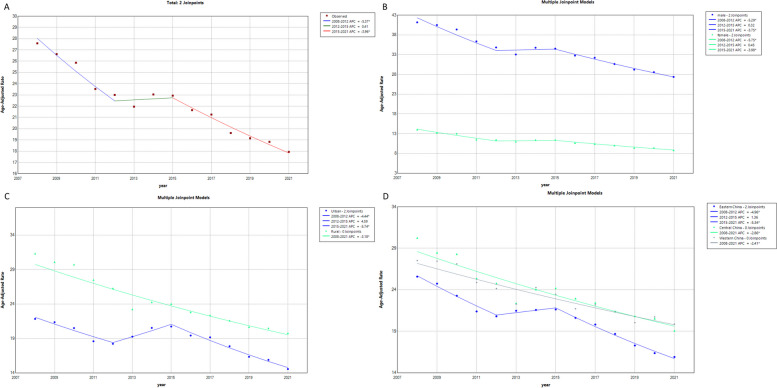
**A** Trend of liver cancer's ASMR from 2008 to 2021 in China(1/100,000). **B** Trends of liver cancer's ASMR from 2008 to 2021 in Male and Female(1/100,000). **C** Trends of liver cancer's ASMR from 2008 to 2021 in Urban and Rural(1/100,000). **D** Trends of liver cancer's ASMR from 2008 to 2021 in Eastern, Central, and Western China(1/100,000)

In 2008, the liver cancer CMR and ASMR in eastern, central, and western China were 25.94/100,000, 25.68/100,000, 26.65/100,000, 30.24/100,000, 23.54/100,000, and 27.51/100,000, respectively. In 2021, the liver cancer CMR and ASMR in eastern, central, and western China were 23.68/100,000, 15.91/100,000, 26.34/100,000, 19.08/100,000, 24.99/100,000, and 19.87/100,000, respectively. The AAPCs of liver cancer CMR and ASMR in eastern, central, and western China were -0.90% (t = -1.00, *P* = 0.30), -3.70% (t = -6.90, *P* < 0.001), 0.30% (t = 1.10, *P* = 0.28), -2.90% (t = -9.50, *P* < 0.001), 0.30% (t = 1.00, *P* = 0.35), and -2.40% (t = -9.10, *P* < 0.001), respectively. There was no significant difference in the AAPC of liver cancer CMR among eastern, central, and western China. However, the liver cancer ASMR in eastern, central, and western China decreased at average annual rates of 3.70%, 2.90%, and2.40%, respectively. Except for 2014, the mortality rates of liver cancer in eastern, central, and western China from 2008 to 2021 were not equal, and the difference was statistically significant (*P* < 0.001). There were no significant differences in liver cancer mortality between eastern and central China in 2008, 2009, 2014, 2015, and 2016. However, in other years, mortality in eastern China was lower than that in central China (*P* < 0.05). There were no significant differences in liver cancer mortality between eastern and western China in 2010, 2011, 2012, 2014, and 2019. However, in other years, mortality in western China was lower than that in eastern China (*P* < 0.05). There were no statistical differences in liver cancer mortality between central and western China in 2013 and 2014. However, in other years, mortality in western China was lower than that in central China (*P* < 0.001). (Table 1 and Fig. 1).

**Table 1:** CMR and ASMR of liver cancer from 2008 to2021 in China (1/100,000)

	Index		2008	2009	2010	2011	2012	2013	2014	2015	2016	2017	2018	2019	2020	2021	AAPC(%) (95%CI)	t	P
Gender	Male	CMR	36.95	37.08	36.21	35.16	35.49	35.86	38.19	37.99	37.07	36.60	35.58	35.48	35.96	35.65	-0.50	-0.60	0.55
		ASMR	41.16	40.48	39.34	36.34	34.81	33.06	34.77	34.55	32.77	32.23	30.64	29.43	28.58	27.37	-3.3(-4.2,-2.4)	-7.20	< 0.001
	Female	CMR	13.59	13.03	12.91	12.32	12.89	13.08	13.77	13.79	13.57	13.36	13.35	13.04	13.79	13.84	0.30	1.60	0.13
		ASMR	14.06	13.24	13.03	11.50	11.45	11.02	11.42	11.50	10.67	10.44	10.04	9.50	9.45	8.85	-3.5(-4.9,-2.1)	-4.90	< 0.001
		χ2	3949	4287	4211	4218	4039	11,938	14,420	14,462	1432	14,545	13,642	14,267	115,645	12,788			
		P	< 0.001	< 0.001	< 0.001	< 0.001	< 0.001	< 0.001	< 0.001	< 0.001	< 0.001	< 0.001	< 0.001	< 0.001	< 0.001	< 0.001			
Area	Urban areas	CMR	22.98	22.79	21.58	19.43	19.63	22.68	23.64	23.95	22.76	22.62	21.84	21.46	21.88	20.79	-0.90	-0.90	0.35
		ASMR	21.83	21.35	20.49	18.57	18.21	19.27	20.54	20.74	19.43	19.17	17.87	16.46	15.89	14.57	-3.0(-4.7,-1.3)	-3.50	< 0.001
	Rural areas	CMR	26.89	26.69	26.75	26.92	27.56	25.59	27.43	27.09	26.93	26.48	26.13	25.98	26.77	27.07	-0.10	-0.40	0.72
		ASMR	31.32	30.10	29.71	27.50	26.27	23.26	24.28	24.30	22.83	22.38	21.60	20.84	20.50	19.78	-3.2(-3.7,-2.6)	-12.50	< 0.001
		χ2	101	103	200	435	478	332	302	213	400	360	456	520	605	956			
		P	< 0.001	< 0.001	< 0.001	< 0.001	< 0.001	< 0.001	< 0.001	< 0.001	< 0.001	< 0.001	< 0.001	< 0.001	< 0.001	< 0.001			
Region	Eastern China	CMR	25.94	25.67	24.17	23.84	23.64	26.18	26.40	26.52	26.03	25.20	24.44	23.71	23.53	23.68	-0.90	-1.00	0.30
		ASMR	25.68	24.73	23.26	21.37	20.79	21.48	21.58	21.63	20.62	19.82	18.69	17.42	16.37	15.91	-3.7(-4.7,-2.7)	-6.90	< 0.001
	Central China	CMR	26.65	25.93	26.26	24.76	25.58	23.87	26.23	26.53	26.22	25.93	25.49	25.93	26.63	26.34	0.30	1.10	0.28
		ASMR	30.24	28.47	28.29	25.43	24.77	22.39	24.05	21.63	22.96	22.44	21.40	20.99	20.54	19.08	-2.9(-3.5,-2.2)	-9.50	< 0.001
	Western China	CMR	23.54	23.99	23.85	22.98	23.95	23.58	25.93	25.01	23.89	24.11	23.89	23.57	25.28	24.99	0.30	1.00	0.35
		ASMR	27.51	27.44	27.10	24.86	24.14	22.28	24.28	23.47	21.72	22.20	21.56	20.20	20.73	19.87	-2.4(-3.0,-1.8)	-9.10	< 0.001
		χ2	45.88	19.60	35.70	15.64	24.16	130.52	3.33	42.32	100.77	53.22	45.45	132.26	197.55	138.9			
		P	< 0.001	< 0.001	< 0.001	< 0.001	< 0.001	< 0.001	> 0.05	< 0.001	< 0.001	< 0.001	< 0.001	< 0.001	< 0.001	< 0.001			
	Eastern China	CMR	25.94	25.67	24.17	23.84	23.64	26.18	26.40	26.52	26.03	25.20	24.44	23.71	23.53	23.68			
		ASMR	25.68	24.73	23.26	21.37	20.79	21.48	21.58	21.63	20.62	19.82	18.69	17.42	16.37	15.91			
	Central China	CMR	26.65	25.93	26.26	24.76	25.58	23.87	26.23	26.53	26.22	25.93	25.49	25.93	26.63	26.34			
		ASMR	30.24	28.47	28.29	25.43	24.77	22.39	24.05	21.63	22.96	22.44	21.40	20.99	20.54	19.08			
		χ2	2.60	0.36	25.18	5.02	21.75	91.08		0.15	0.67	10.68	22.37	100.97	196.19	138.84			
		P	> 0.05	> 0.05	< 0.001	< 0.05	< 0.001	< 0.001		> 0.05	> 0.05	< 0.05	< 0.001	< 0.001	< 0.001	< 0.001			
	Eastern China	CMR	25.94	25.67	24.17	23.84	23.64	26.18	26.40	26.52	26.03	25.20	24.44	23.71	23.53	23.68			
		ASMR	25.68	24.73	23.26	21.37	20.79	21.48	21.58	21.63	20.62	19.82	18.69	17.42	16.37	15.91			
	Western Chian	CMR	23.54	23.99	23.85	22.98	23.95	23.58	25.93	25.01	23.89	24.11	23.89	23.57	25.28	24.99			
		ASMR	27.51	27.44	27.10	24.86	24.14	22.28	24.28	23.47	21.72	22.20	21.56	20.20	20.73	19.87			
		χ2	27.24	13.56	0.51	3.84	0.48	93.33		36.17	75.44	20.22	5.20	0.35	55.56	30.29			
		P	< 0.001	< 0.001	> 0.05	> 0.05	> 0.05	< 0.001		< 0.001	< 0.001	< 0.001	< 0.05	> 0.05	< 0.001	< 0.001			
	Central China	CMR	26.65	25.93	26.26	24.76	25.58	23.87	26.23	26.53	26.22	25.93	25.49	25.93	26.63	26.34			
		ASMR	30.24	28.47	28.29	25.43	24.77	22.39	24.05	21.63	22.96	22.44	21.40	20.99	20.54	19.08			
	Western Chian	CMR	23.54	23.99	23.85	22.98	23.95	23.58	25.93	25.01	23.89	24.11	23.89	23.57	25.28	24.99			
		ASMR	27.51	27.44	27.10	24.86	24.14	22.28	24.28	23.47	21.72	22.20	21.56	20.20	20.73	19.87			
		χ2	45.26	17.22	27.46	15.40	12.29	1.18		30.74	85.83	53.22	41.57	91.77	29.29	28.08			
		P	< 0.001	< 0.001	< 0.001	< 0.001	< 0.001	> 0.05		< 0.001	< 0.001	< 0.001	< 0.001	< 0.001	< 0.001	< 0.001			
	Total	CMR	25.52	25.30	24.79	23.94	24.40	24.69	26.22	26.08	25.53	25.17	24.66	24.43	25.05	24.92	-0.10	-0.70	0.47
		ASMR	27.58	26.63	25.85	23.53	22.99	21.98	23.06	22.95	21.67	21.27	20.29	19.31	18.84	17.95	-3.4(-4.7,-2.1)	-5.10	< 0.001

### Burden and trends of liver cancer death

**Fig. 2:**
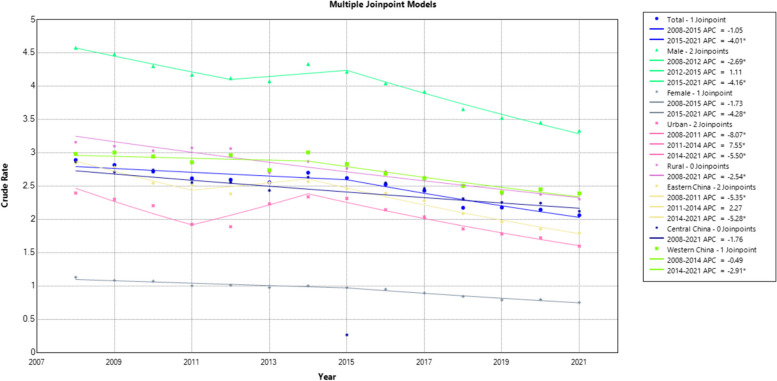
Trend of liver cancer's PYLLR from 2008 to 2021 in China(‰)

In 2008 and 2021, the PYLLR of liver cancer in Chinese residents was 2.89‰ and 2.06‰, respectively, and its AAPC was -2.40% (t = -5.10, *P* < 0.001). In 2008 and 2021, the PYLLR of male liver cancer was 4.58‰ and 3.33‰, respectively, and its AAPC was -2.20% (t = -5.4, *P* < 0.001). In 2008 and 2021, the PYLLR of female liver cancer was 1.13‰ and 0.75‰, respectively, and its AAPC was -2.90% (t = -8.40, *P* < 0.001). The PYLLR of liver cancer from 2008 to 2021 was higher in males than in females. In 2008 and 2021, the PYLLR of urban liver cancer was 2.39‰ and 1.60‰, respectively, and its AAPC was -3.30% (t = -4.00, *P* < 0.001). In 2008 and 2021, the PYLLR of rural liver cancer was 3.16‰ and 2.30‰, respectively, and its AAPC was -2.50% (t = -11.60, *P* < 0.001). The PYLLR of liver cancer from 2008 to 2021 was higher in rural areas than in urban areas. In 2008 and 2021, the PYLLR of liver cancer in eastern China was 2.85‰ and 1.79‰, respectively, and its AAPC was -3.40% (t = -7.80, *P* < 0.001). In 2008 and 2021, the PYLLR of liver cancer in central China was 2.87‰ and 2.12‰, respectively, and its AAPC was -2.30% (t = -3.60, *P* < 0.001). In 2008 and 2021, the PYLLR of liver cancer in western China was 2.98‰ and 2.39‰, respectively, and its AAPC was -2.1% (t = -7.1, *P* < 0.001). The annual PYLLR of liver cancer in western China was higher than that in eastern and central China from 2008 to 2021. Except for 2009 and 2013, the annual PYLLR of liver cancer in central China was higher than that in eastern China from 2008 to 2021. The PYLLR of liver cancer in China, males, females, urban, rural, eastern, central, and western China decreased at an average annual rate of 2.40%, 2.20%, 2.90%, 3.30%, 2.50%, 3.40%, 2.30%, 2.10%, respectively (*P* < 0.001). The rate of decline was higher in females than in males, higher in urban areas than in rural areas, and higher in eastern China than in central and western China. (Table 2 and Fig. 2).

**Table 2:** PYLL and PYLLR of liver cancer from 2008 to 2021 in China (1/100,000)

Index		2008	2009	2010	2011	2012	2013	2014	2015	2016	2017	2018	2019	2020	2021	AAPC(%) (95%CI)	t	P
Male	PYLL	173,293	171,454	172,704	164,266	161,994	471,629	560,167	551,331	.544801.5	539,655	505,961	505,961	488,379	453,076			
	PYLLR (‰)	4.59	4.48	4.30	4.17	4.12	4.07	4.33	4.22	4.04	3.92	3.65	3.65	3.45	3.3305	-2.2(-3.0,-1.4)	-5.4	< 0.001
Female	PYLL	40,862	39,769	41,334	38,074	38,309	108,725	124,407	123,434	123,381	119,031	112,639	112,639	108,848	98,962			
	PYLLR (‰)	1.13	1.08	1.07	1.00	1.01	0.98	1.00	0.97	0.95	0.89	0.84	0.84	0.79	0.7505	-2.9(-3.6,-2.2)	-8.4	< 0.001
Urban areas	PYLL	61,930	61,341	65,709	59,385	58,113	156,760	189,209	191,873	189,622	186,340	172,571	172,571	168,175	146,490			
	PYLLR (‰)	2.39	2.30	2.20	1.92	1.89	2.23	2.34	2.31	2.14	2.03	1.86	1.86	1.72	1.5963	-3.3(-4.8,-1.7)	-4	< 0.001
Rural areas	PYLL	151,955	149,882	148,329	142,966	142,190	423,595	495,365	482,891	478,561	472,346	446,040	446,040	429,052	405,547			
	PYLLR (‰)	3.16	3.10	3.03	3.07	3.06	2.70	2.87	2.77	2.71	2.63	2.49	2.49	2.37	2.3026	-2.5(-3.0,-2.1)	-12	< 0.001
Eastern China	PYLL	80,304	79,760	78,195	75,734	71,574	223,569	255,584	243,151	244,428	243,377	223,517	223,517	202,672	192,272			
	PYLLR (‰)	2.85	2.78	2.54	2.51	2.38	2.55	2.56	2.46	2.39	2.28	2.09	2.09	1.85	1.7932	-3.4(-4.2,-2.6)	-7.8	< 0.001
Central China	PYLL	72,831	70,205	73,069	69,036	69,215	200,590	236,047	235,237	237,296	233,598	218,582	218,582	213,688	190,138			
	PYLLR (‰)	2.87	2.71	2.74	2.55	2.56	2.43	2.63	2.63	2.55	2.46	2.31	2.31	2.24	2.1208	-2.3(-3.5,-1.0)	-3.6	< 0.001
Western China	PYLL	60,521	61,259	62,775	57,571	59,514	156,196	192,944	196,727	186,459	181,712	176,501	176,501	180,867	169,628			
	PYLLR (‰)	2.98	3.00	2.94	2.86	2.96	2.74	3.00	2.83	2.68	2.62	2.50	2.50	2.45	2.3885	-2.1(-2.7,-1.4)	-7.1	< 0.001
Total	PYLL	213,655	211,223	214,038	202,340	200,303	580,354	684,574	674,764	6,668,182	658,686	591,600	591,600	597,227	552,037			
	PYLLR (‰)	2.89	2.82	2.72	2.61	2.59	2.55	2.70	2.62	2.52	2.43	2.17	2.17	2.14	2.0607	-2.4(-3.4,-1.5)	-5.1	< 0.001

### The age-period-cohort model analysis of China liver cancer mortality

**Fig. 3:**
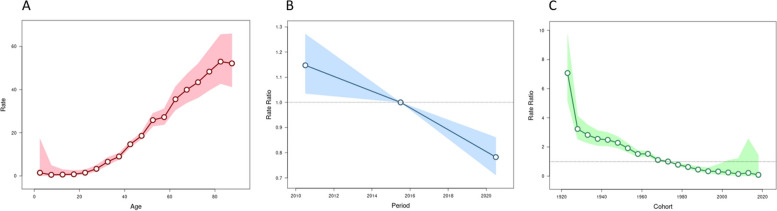
**A** Age effect on China liver cancer mortality. **B** Period effect on China liver cancer mortality. **C** Cohort effect on China liver cancer mortality

After controlling for period and cohort effects, the age effect on liver cancer showed that mortality risk continuously increased with advancing age, peaked at 81–85 years old. The period effect on liver cancer presented that mortality risk decreased from 2008 to 2021, which was consistent with the result of the Annual-percent-change (APC). The cohort effect on liver cancer showed that mortality risk continuously decreased from the earlier birth cohort to the later birth cohort. Before the 1971–1975 birth cohort, the risk was higher than the overall average, it was opposite after 1971–1975 birth cohort. (Fig. 3 & Table S2).

### Trends in the sex ratio of liver cancer mortality with age

**Fig. 4:**
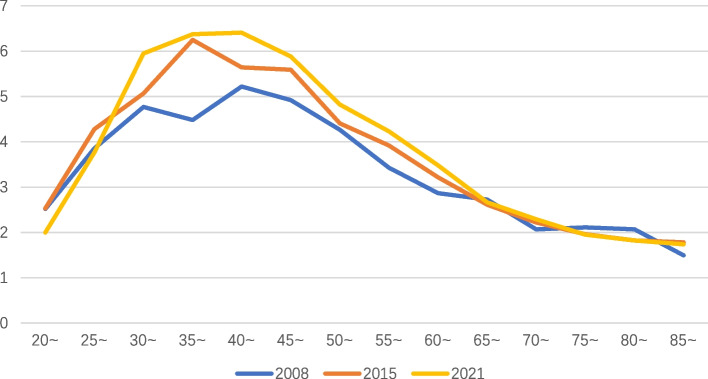
Trend of the sex ratio of liver cancer mortality rate (male: female) with age in China

Before the age of 20, the mortality rate of liver cancer is too low. Therefore, the change in the sex ratio of mortality with age is analyzed from the age of 20. The male to female ratio of the liver cancer mortality rate gradually increased from 2 ~ 3 times at the age of 20 to 5 ~ 6.6 times at the age of 40 and then declined to 1 ~ 2 times at 85 years old. (Fig. 4).

## Discussion

This article analyzed the trends of liver cancer mortality and disease burden in Chinese residents from 2008 to 2021.

In 2021, the ASMR of liver cancer in China was 17.95/100,000, which is higher than the global standardized rate of 8.7/100,000. The ASMR for males was 27.37/100,000 and for females was 8.85/100,000, both higher than the global standardized rates of 12.9/100,000 and 4.8/100,000, respectively [13]. This may be related to the higher incidence of hepatitis B infection, per capita alcohol consumption, aflatoxin exposure levels, nonalcoholic fatty liver disease (NAFLD), and diabetes in China. Hepatitis B remains a major worldwide public health problem, with approximately 257 million individuals infected with the hepatitis B virus (HBV) and over 94 million having chronic hepatitis B [14]. There are approximately 120 million hepatitis B virus carriers in China, and 30 million people are chronically infected with the hepatitis B virus, accounting for nearly one-third of the world's hepatitis B virus infections [15]. In 2016, the average intake of pure alcohol among drinkers over 15 years old in China was 12.7L [16]. The average alcohol consumption among the Chinese population (7.2 L of pure alcohol) was 12.5% higher than the global average (6.4 L) [17]. In China, the rates of aflatoxin contamination in corn and peanuts were as high as 70.27% and 24.24%, respectively [18]. The current prevalence of NAFLD is approximately 25.24% globally (95% CI: 22.10–28.65) [19], and it is approximately 29.2% (95% CI: 27.7–30.7) in China [20]. In 2017, there were 476 million diabetes patients worldwide, of which approximately 89.496 million were in China [21]. Currently, chronic HBV infection, chronic HCV infection, and NAFLD are the most important pathogenic factors of liver cancer. Alcoholic liver disease, obesity, and diabetes also increase the risk of liver cancer [19]. Aflatoxin exposure, smoking, heavy alcohol consumption, low vegetable intake, radioactive thorium dioxide exposure, iron overload, oral contraceptives, and steroids are thought to be risk factors for hepatocellular carcinoma (HCC) [22]. Among them, chronic HBV infection has a high incidence of 50%-80% in hepatocellular carcinoma (HCC) [23].

From 2008 to 2021, the ASMR of liver cancer was lower than the CMR, indicating that the mortality rate of liver cancer in the elderly population was higher. And the Age-Period-Cohort also presented it. This is consistent with the conclusion of Jia-Ying Fang et al. [24].

liver cancer mortality risk increases gradually with age and peaked at 81–85 years. Age-specific incidence may be related to the type of hepatitis virus predominant in different populations, age of infection, and other risk factors [25]. The higher mortality rate of liver cancer in the elderly population may be related to older age, worse health, more underlying diseases, and longer exposure to risk factors. In addition, chronic diseases in the elderly can also increase the risk of liver cancer [26].

From 2008 to 2021, the ASMRs of liver cancer in China and in male, female, urban, rural, eastern, central and western residents decreased at average annual rates of 3.40%, 3.30%, 3.50%, 3.00%, 3.20%, 3.70%, 2.90%, 2.40%, respectively (*P* < 0.001). This was associated with reduced seroprevalence of HBV and HCV in the population, reduced aflatoxin exposure, and hepatitis B vaccination [27]. It may also be related to the improvement of the economy and the increase in residents' health awareness.

From 2008 to 2021, the mortality rate of liver cancer in males was 2 ~ 3 times (*P* < 0.001) higher than that in females. Studies have found that estrogen has a certain protective effect on the development of HCC in the physiological state of premenopausal females [28]. Li Peng et al. [29] found that sex differences and expression levels of AR/ER can affect the prognosis of HCC patients, and male sex is associated with a poor prognosis. It is also associated with more risk-factor exposure in males: males are more susceptible to HBV and HCV, smoking, alcohol consumption, increased iron stores and androgens, and obesity [30].

From 2008 to 2021, the mortality rate of liver cancer in rural areas was higher than that in urban areas (*P* < 0.001). The high mortality rate for primary liver cancer in rural areas may be due to differences with urban areas in educational attainment, household economic income, pesticide exposure, and availability of and adherence to antiviral therapy [31]. Rural areas have a high incidence of viral hepatitis and are more susceptible to aflatoxin exposure, and the incidence of liver cancer is also high [32].

From 2008 to 2021, Eastern China had the lowest ASMR for liver cancer, followed by Central and Eastern China. Studies have shown that the incidence of liver cancer is highest in Western China, followed by Central China, and lowest in Eastern China [33]. There may be differences in the case fatality rate of liver cancer in the Eastern, Central and Western regions. According to an epidemiological survey in a high-incidence area, factors such as diet and food contaminated by Aspergillus flavus, as well as increased nitrosamine content, may be related to the cause of the high incidence [34]. The lowest ASMR of liver cancer in Eastern China may be related to the region's relatively developed economy, high vaccination rates for hepatitis B in neonates, low levels of aflatoxin exposure, and strong public health awareness.

From 2008 to 2021, the PYLLR of liver cancer in China and in male, female, urban, rural, eastern, central, and western residents decreased at an average annual rate of2.40%, 2.20%, 2.90%, 3.30%, 2.50%, 3.40%, 2.30%, 2.10%, respectively. This suggests that the disease burden of liver cancer death is decreasing year by year, which is related to China's long-term effective neonatal HBV vaccination policy, the reduction of aflatoxin contamination in the diet, and alpha-fetoprotein screening in high-incidence areas [35]. The Age-Period-Cohort showed that after 1971–1975 birth cohort, the liver cancer mortality risk was lower than the overall average. Considering that Chinese government had launched a hepatitis B vaccine immunization program since 1985 [36], it is reasonable to infer that hepatitis B vaccination further reduced the risk of liver cancer mortality, but the correlation between liver cancer mortality and hepatitis B vaccination rate still needs further research.

From 2008 to 2021, the PYLLR of liver cancer was higher in males than in females, higher in rural areas than in urban areas, and higher in western than in eastern and central China. This suggests that liver cancer has brought a heavier disease burden to male, rural, and western regions in China. This is similar to the conclusions of Jiang Nan et al. [37], who concluded that from 2005 to 2009, the disease burden of liver cancer in China's tumor registration areas was still relatively heavy, with males having a higher incidence than females and people living in rural areas having a higher incidence than those living in urban areas.

We also found that PYLLR and AMSR of liver cancer continued to decline from 2008–2021, except for 2012–2015, especially in urban areas (Fig. 1 & Fig. 2). This may be related to China’s launch of the China Chronic Disease Prevention and Control Work Plan (2012–2015), which proposed the goals: early diagnosis and treatment of priority cancers should be carried out in 30% of the cancer-prone-areas, the Cause-of-Death Surveillance dataset should cover 90% of counties nationwide [38]. This is also proved by the increase in the surveillance population in the cause-of-death surveillance dataset from 77,215,997 in 2012 to 227,236,284 in 2013. The expansion of the early cancer screening and surveillance population may led to an increased detection rate of liver cancer, resulting in the paradoxical changes in the PYLLR and AMSR of liver cancer from 2012 to 2015. In 2012, the National Health Commission launched a major national public health service project—Early Diagnosis and Treatment of Urban Cancer [39]. From 2013 to 2017, 2,679,670 people were surveyed, 1,140,854 people were evaluated as high-risk groups, and 732,974 medical examinations for high-incidence cancers were completed in high-risk groups, with a detection rate of 2.18% for liver cancer [40]. Early diagnosis and treatment of urban cancers resulted the increased detection rate of liver cancer in urban areas. This may contributed to the more pronounced paradoxical changes in the PYLLR and AMSR of liver cancer in urban areas from 2012 to 2015.

From 2008 to 2021, the mortality rate of liver cancer in males was higher than that in females by approximately 2 ~ 3 times, and there was an age distribution difference in the male to female ratio for liver cancer mortality: approximately 2 ~ 3 times at 20 years old, approximately 5 ~ 6 times at 40 years old, and approximately 1 ~ 2 times at 85 years old, which may be related to the protective effect of estrogen and the decline in estrogen levels in perimenopausal females.

Therefore, liver cancer prevention and treatment in China should focus on male, rural, western, and elderly residents. More attention should be given to the primary prevention of liver cancer, including immunization with HBV vaccines, antiviral therapies to the patients with chronic hepatitis B or hepatitis C, avoiding or reducing the exposure to aflatoxins as well as the cyanotoxins, quitting smoking and limiting alcohol consumption, etc. [41]. Policies and resources for the primary prevention of liver cancer should be tilted towards male, rural, western, and elderly residents. Thus, to reduce the mortality and disease burden of liver cancer.

### Supplementary Information

Supplementary material 1.

Supplementary material 2.

## Data Availability

Data are available in a public, open access repository. All data used are publicly available from the National Cause-of-Death Surveillance System (https://ncncd.chinacdc.cn/xzzq_1/202101/t20210111_223706.htm).

## Abbreviations

CMR: Crude Mortality Rate
ASMR: Age-Standardized Mortality Rate
PYLL: Potential Years of Life Lost
PYLLR: Potential Years of Life Lost Rate
APC: Annual percent change
